# Closed-loop glucose control in young people with type 1 diabetes during and after unannounced physical activity: a randomised controlled crossover trial

**DOI:** 10.1007/s00125-017-4395-z

**Published:** 2017-08-24

**Authors:** Klemen Dovc, Maddalena Macedoni, Natasa Bratina, Dusanka Lepej, Revital Nimri, Eran Atlas, Ido Muller, Olga Kordonouri, Torben Biester, Thomas Danne, Moshe Phillip, Tadej Battelino

**Affiliations:** 1 Department of Paediatric Endocrinology, Diabetes and Metabolic Diseases, University Children’s Hospital, University Medical Centre Ljubljana, Bohoriceva 20, SI–1000 Ljubljana, Slovenia; 2 Department of Paediatrics–Diabetes Service Studies, University of Milan, Ospedale dei Bambini Vittore Buzzi, Milan, Italy; 3 Department of Pulmonology, University Children’s Hospital, University Medical Centre Ljubljana, Ljubljana, Slovenia; 4 The Jesse and Sara Lea Shafer Institute for Endocrinology and Diabetes, National Centre for Childhood Diabetes, Schneider Children’s Medical Centre of Israel, Petah Tikva, Israel; 5 DreaMed Diabetes Ltd, Petah Tikva, Israel; 6 Diabetes Centre for Children and Adolescents, Kinder- und Jugendkrankenhaus Auf der Bult, Hannover, Germany; 7 Sackler Faculty of Medicine, Tel Aviv University, Tel Aviv, Israel; 8 Faculty of Medicine, University of Ljubljana, Ljubljana, Slovenia

**Keywords:** Clinical science, Devices, Diabetes in childhood, Exercise, Hypoglycaemia

## Abstract

**Aims/hypothesis:**

Hypoglycaemia during and after exercise remains a challenge. The present study evaluated the safety and efficacy of closed-loop insulin delivery during unannounced (to the closed-loop algorithm) afternoon physical activity and during the following night in young people with type 1 diabetes.

**Methods:**

A randomised, two-arm, open-label, in-hospital, crossover clinical trial was performed at a single site in Slovenia. The order was randomly determined using an automated web-based programme with randomly permuted blocks of four. Allocation assignment was not masked. Children and adolescents with type 1 diabetes who were experienced insulin pump users were eligible for the trial. During four separate in-hospital visits, the participants performed two unannounced exercise protocols: moderate intensity (55% of $\overset{\cdot }{V}{\mathrm{O}}_{2\max }$) and moderate intensity with integrated high-intensity sprints (55/80% of $\overset{\cdot }{V}{\mathrm{O}}_{2\max }$), using the same study device either for closed-loop or open-loop insulin delivery. We investigated glycaemic control during the exercise period and the following night. The closed-loop insulin delivery was applied from 15:00 h on the day of the exercise to 13:00 h on the following day.

**Results:**

Between 20 January and 16 June 2016, 20 eligible participants (9 female, mean age 14.2 ± 2.0 years, HbA_1c_ 7.7 ± 0.6% [60.0 ± 6.6 mmol/mol]) were included in the trial and performed all trial-mandated activities. The median proportion of time spent in hypoglycaemia below 3.3 mmol/l was 0.00% for both treatment modalities (*p* = 0.7910). Use of the closed-loop insulin delivery system increased the proportion of time spent within the target glucose range of 3.9–10 mmol/l when compared with open-loop delivery: 84.1% (interquartile range 70.0–85.5) vs 68.7% (59.0–77.7), respectively (*p* = 0.0057), over the entire study period. This was achieved with significantly less insulin delivered via the closed-loop (*p* = 0.0123).

**Conclusions/interpretation:**

Closed-loop insulin delivery was safe both during and after unannounced exercise protocols in the in-hospital environment, maintaining glucose values mostly within the target range without an increased risk of hypoglycaemia.

**Trial registration:**

Clinicaltrials.gov NCT02657083

**Funding:**

University Medical Centre Ljubljana, Slovenian National Research Agency, and ISPAD Research Fellowship

**Electronic supplementary material:**

The online version of this article (doi:10.1007/s00125-017-4395-z) contains peer-reviewed but unedited supplementary material, which is available to authorised users.

## Introduction

Regular physical activity is a fundamental part of type 1 diabetes management recommendations [1] and has a positive impact on cardiovascular health, insulin requirement, body fitness and general wellbeing [2]. Recent data from large diabetes registries have shown a positive correlation between physical activity and metabolic control [3].

The physical capacity of young people with diabetes and their healthy peers is comparable [4]; however, physical activity is often associated with an increased risk of glycaemic excursions and hypoglycaemia, particularly during the activity and the night after it [4, 5]. Several strategies have been suggested for limiting this risk, including recommendations on basal insulin adjustments and additional carbohydrate consumption, although these recommendations are inconsistent and based on limited evidence, particularly for the paediatric population [6–8]. The incorporation of intermittent high-intensity sprints into moderate exercise is associated with less hypoglycaemia [9] and is recommended by the International Society for Paediatric and Adolescent Diabetes (ISPAD) as a possible strategy to minimise the risk for hypoglycaemia [10].

Closed-loop insulin delivery systems have recently been demonstrated to be safe and efficient in summer camps [11] and free-living conditions [12–16]. Data on closed-loop insulin delivery during physical activity is scarce [17, 18], particularly in children and adolescents. There have been reports of no increase in the frequency of post-exercise nocturnal hypoglycaemia and overall hypoglycaemia frequency being comparable [19] or lower [20] during closed-loop delivery when compared with open-loop delivery as control.

The present randomised controlled study investigated glucose control under open-loop and closed-loop insulin delivery, during and after unannounced afternoon moderate physical activity with or without intermittent high-intensity sprints, in young people with type 1 diabetes.

## Methods

### Study design and participants

This open-label, randomised, two-arm, crossover, in-hospital clinical trial was conducted at the University Children’s Hospital in Ljubljana, Slovenia. The study was performed in compliance with the Declaration of Helsinki, Good Clinical Practice, and applicable regulatory requirements. The Slovenian National Medical Ethics Committee and the regulatory authority approved the protocol. All participants and their parents provided written informed assent/consent before trial initiation. The study is listed on clinicaltrials.gov under the registration number NCT02657083.

The inclusion criteria were: age 10–17 years (inclusive), clinical diagnosis of type 1 diabetes for at least 1 year, at least 3 months of current use of an insulin pump, HbA_1c_ below 9.0% (75 mmol/mol), BMI within normal range for age and sex (± 2 SD) and the absence of other medical conditions (apart from well controlled hypothyroidism or coeliac disease). Exclusion criteria included concomitant diseases that could influence metabolic control or compromise a participant’s safety, known hypoglycaemia unawareness or more than two episodes of severe hypoglycaemia with seizure and/or coma within the 6 months prior to the screening, and history of one or more episodes of diabetic ketoacidosis requiring hospitalisation within 1 month prior to the screening.

**Fig. 1:**
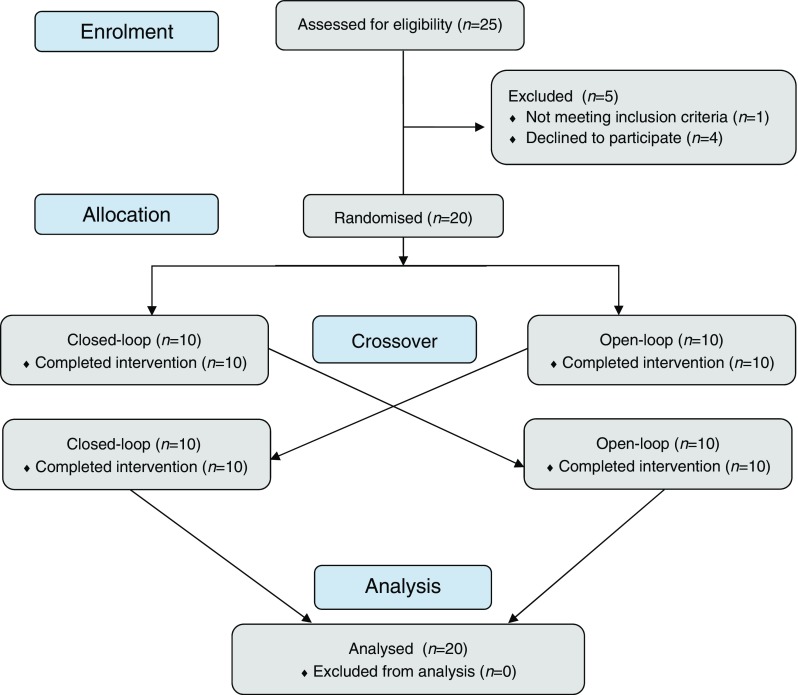
Study flow diagram. Schedule for all sessions was the same: 13:00 h, lunch; 16:00 h, snack; 16:30–19:30 h, exercise time; 19:45 h, dinner; next day 8:00 h, breakfast; 13:00, lunch

After screening and baseline visits (visits 1 and 2), the study included four 24 h in-hospital sessions for each participant (visits 3–6) (Fig. 1). The observation period was defined as the time between 15:00 h on day 1 and 13:00 h the next day. To detect hypoglycaemia on the day following exercise we prolonged our observational period until 13:00 h the next day. During this time, the participants performed one of the two exercise protocols using either a closed-loop or open-loop insulin delivery device. There was 1 week in between each session and 1 week between different study arms.

### Randomisation and masking

Participants were randomly assigned (1:1) to perform physical activity on two consecutive days using closed-loop insulin delivery followed by physical activity on 2 days using an insulin pump with glucose sensor and without computer algorithm (open-loop), or vice versa. Following the run-in period, the order was randomly determined using an automated web-based programme with randomly permuted blocks of four. Participants and investigators analysing study data were not masked to treatment.

### Procedures

The screening visit (visit 1) included informed consent acquisition, detailed physical examination and confirmation of inclusion/exclusion criteria. Participants were trained in the use of the glucose sensor before entering a run-in period. At the baseline visit (visit 2) all participants performed a lung function test, resting ECG and a cycle ergometer exercise test to determine their maximal oxygen consumption rate ($\overset{\cdot }{V}{\mathrm{O}}_{2\max }$). We downloaded the run-in period (at least 5 days) glucose sensor data to derive the initial personalised closed-loop system settings. The insulin dose during the in-hospital stay was decided by a trained nurse educator in consultation with the participant and, if in doubt, with the on-call diabetologist. On the basis of the insulin pump download, we excluded participants with a hypoglycaemic event (glucose level below 2.8 mmol/l) on the day before the intervention, as they could be at increased risk of hypoglycaemia during exercise.

Participants were instructed to insert the sensor on the day before their hospital visit. Upon admission, a nurse educator checked the sensor and a backup sensor was inserted when in doubt. During the in-hospital stay, sensor calibration was scheduled three times: at initiation of the closed-loop insulin delivery system (around 15:00 h on day 1), before dinner (from 20:00 to 21:00 h) and in the morning of day two (around 7:00 h). If the glucose level was out of range or there was discrepancy between blood and sensor glucose, the calibration was postponed until the glucose level was stable.

During separate in-hospital visits (visits 3–6), participants performed two different 40 min protocols of afternoon physical activity (exercise was started between 16:30 and 19:30 h) on a cycle ergometer. The moderate intensity (55% $\overset{\cdot }{V}{\mathrm{O}}_{2\max }$) physical activity protocol and a combination of moderate physical activity with incorporated high-intensity (80% $\overset{\cdot }{V}{\mathrm{O}}_{2\max }$) sprints (55/80% $\overset{\cdot }{V}{\mathrm{O}}_{2\max }$ protocol) were carried out under closed-loop or open-loop insulin delivery, in random order. In both protocols, participants were instructed to pedal at a steady rate of 50–60 rev/min for 40 min, at 55% $\overset{\cdot }{V}{\mathrm{O}}_{2\max }$ load (starting workload set at 30 watts with linear loading to reach 55% $\overset{\cdot }{V}{\mathrm{O}}_{2\max }$ at 5 min exercise time; the load based on $\overset{\cdot }{V}{\mathrm{O}}_{2\max }$ was adjusted in real time as needed). In the 55/80% $\overset{\cdot }{V}{\mathrm{O}}_{2\max }$ protocol, high-intensity sprints with a duration of 20 s were incorporated, with intervals of 6–10 min activity at 55% $\overset{\cdot }{V}{\mathrm{O}}_{2\max }$ between the sprints, for a total of 40 min.

Throughout all exercises, a continuous ECG was recorded, and inhaled O_2_ and exhaled CO_2_ were measured. Capillary blood glucose was checked at the beginning of each exercise session, every 15 min during the exercise and every 30 min for 2 h after the exercise.

During the hospitalisation, all participants received standardised meals containing approximately 1 g of carbohydrate per kg of body mass for the main meals (lunch, dinner and breakfast) and about half of this amount for the snacks. In both study arms, all meals were covered with manual insulin boluses according to the individual’s carbohydrate-to-insulin ratio. For the open-loop insulin delivery control, the device was disconnected during the exercise and the basal insulin dose was reduced by 20% for 4 h following the exercise session. During the closed-loop insulin delivery, the use of the pump was uninterrupted; the device was applied from 15:00 h on the day of the exercise to 13:00 h on the next day, and exercise was not announced to the closed-loop algorithm.

### Devices and assays

All participants used an identical insulin pump (Paradigm Veo; Medtronic Diabetes, Northridge, CA, USA), a subcutaneous glucose sensor (Enlite II sensor with MiniLink REAL-Time transmitter; Medtronic Diabetes) and a glucose meter (Contour Link meter; Ascensia HealthCare, Basel, Switzerland). The low-glucose threshold insulin suspension function was disabled for all participants.

The closed-loop algorithm (Glucositter; DreaMed Diabetes, Petah Tikva, Israel) used a modified vendor-supplied communication module application programming interface (API) to retrieve glucose/insulin data from the MiniMed Paradigm Veo pump and set insulin treatment according to a fuzzy-logic-based algorithm [21]. The software version 01.05.02 operated on a commercial laptop/tablet computer (ThinkPad T450s; Lenovo, Beijing, China), which had a physical connection to a communication dongle (provided by the manufacturer of the insulin pump). The closed-loop software was implemented using the Matlab platform (MathWorks, Natick, MA, USA).

The closed-loop system requires a patient-specific log file for its operation. This log file includes the treatment settings for an individual that are downloaded from the sensor-augmented insulin pump (SAP) (based on run-in period data—an individual’s sensitivity factor, carbohydrate factor and basal insulin settings). Once this pre-made log file exists inside the closed-loop device (dedicated laptop in this case), for each individual, the physician can launch the application, check and approve the settings and insert the pump serial number. From there, the system automatically connects to the pump and sensor and controls them.

The exercise protocol was performed on a cycle ergometer (Power Cube LF8.5G with Schiller software; Ganshorn, Niederlauer, Germany).

The HbA_1c_ level was determined by an immunochemical method using the Siemens DCA Vantage Analyser (Siemens Healthcare, Erlangen, Germany).

### Safety monitoring

A hypoglycaemic event was defined as a blood glucose level below 3.3 mmol/l based on sensor glucose readings, with a minimum duration of 20 min. For this study, not every hypoglycaemic event was reported as an adverse event. All sensor glucose values under 3.3 mmol/l were recorded by the study device and included in the statistical analysis. Severe hypoglycaemia was considered a serious adverse event and was defined as glucose under 2.8 mmol/l, accompanied by a seizure or loss of consciousness, as per ISPAD guidelines, or if it required intravenous glucose and/or intramuscular glucagon administration.

All sensor glucose-detected hypoglycaemic events were additionally confirmed with self-monitoring of blood glucose (SMBG). When glucose values fell below 3.3 mmol/l if symptomatic, and when glucose values fell below 2.8 mmol/l, regardless of symptoms, 15 g rescue carbohydrates were administered as per standard in-hospital procedures, and recorded as an adverse event.

Hyperglycaemia or diabetic ketoacidosis were considered a serious adverse event only if blood glucose rose above 13.9 mmol/l and was associated with low serum bicarbonate (< 15 mmol/l) or low pH (< 7.3) and either ketonaemia (β-hydroxybutyrate level above 3 mmol/l) or ketonuria requiring intravenous treatment. Other hyperglycaemic events were not reported as adverse events; however, they were recorded by the study device and included in the final analysis.

### Endpoints

The primary endpoint was the difference in time spent in hypoglycaemia below 3.3 mmol/l during the unannounced afternoon exercise and the night after (whole observation period from 15:00 h on the day of exercise to 13:00 h on the following day; overnight time from 22:00 h to 07:00 h the next day), based on sensor glucose readings, with a minimum duration of 20 min. Hypoglycaemic events were confirmed with SMBG.

The secondary endpoints were defined as follows: (1) the proportion of time spent with glucose values of 3.9–10 mmol/l; (2) the proportion of time spent in hypoglycaemia below 3.9 mmol/l; (3) the proportion of time spent in hyperglycaemia above 13.9 mmol/l; and (4) fasting glucose on the morning after the physical activity.

The study endpoints are in line with recommendations for outcome measurements for artificial pancreas clinical trials [22].

### Statistical analysis

Analyses were based on the modified intention-to-treat population, defined as all randomly assigned participants who had more than 67% sensor measurements. Comparisons between closed-loop and open-loop insulin delivery systems were performed using the paired nonparametric Wilcoxon signed rank test. The power of the nonparametric tests for the primary endpoint was based on the results of power simulations (MATLAB 2013b, MathWorks) based on previous studies [11, 23, 24]. We calculated that enrolment of 20 participants would provide a power of 90% for detecting a 30% reduction in the proportion of time spent with blood glucose levels below 3.3 mmol/l, at a 0.05 two-sided significance level, assuming a 30% dropout.

## Results

Between 20 January and 16 June 2016, 25 children and adolescents with type 1 diabetes were invited to participate, through the Slovenian National Diabetes Registry [25], and 20 (9 female) were randomised. They all completed the study and provided data for analysis (study flow diagram is presented in Fig. 1). Baseline characteristics are shown in Table 1. The mean age was 14.2 ± 2.0 years, duration of diabetes 8.3 ± 3.2 years, HbA_1c_ 7.7 ± 0.6% (60.0 ± 6.6 mmol/mol), duration of pump therapy 7.4 ± 3.2 years and the total daily insulin dose 0.8 ± 0.2 U/kg. Participants were of average physical fitness: mean BMI 21.5 ± 4.3 kg/m^2^; $\overset{\cdot }{V}{\mathrm{O}}_{2\max }$ 43.3 ± 9.3 ml kg^˗1^ min^˗1^ (36.1 ± 4.0 ml kg^˗1^ min^˗1^ for girls and 49.2 ± 8.1 ml kg^˗1^ min^˗1^ for boys) and maximal heart rate 186.6 ± 10.2 beats/min (182.9 ± 11.9 beats/min for girls and 189.6 ± 7.9 beats/min for boys). The amount of carbohydrate consumed during the study period was 1.14 ± 0.34 g/kg for main meals and 0.55 ± 0.41 g/kg for snacks.

**Table 1:** Baseline characteristics of participants

Characteristic	All (*n* = 20)	Male sex (*n* = 11)	Female sex (*n* = 9)
Age (years)	14.2 ± 2.0	13.7 ± 2.0	14.9 ± 2.0
Duration of diabetes (years)	8.3 ± 3.2	7.9 ± 2.7	8.7 ± 3.8
Duration using pump (years)	7.4 ± 3.2	7.0 ± 2.8	7.9 ± 3.8
BMI (kg/m^2^)	21.5 ± 4.3	19.2 ± 3.0	24.4 ± 4.2
BMI SDS (percentile)	63.6 ± 26.9	53.3 ± 27.1	76.2 ± 21.8
HbA_1c_ (%)	7.7 ± 0.6	7.5 ± 0.5	7.9 ± 0.7
HbA_1c_ (mmol/mol)	60 ± 6.6	58.5 ± 5.5	62.8 ± 7.7
Total daily insulin (U/kg)	0.8 ± 0.2	0.8 ± 0.2	0.8 ± 0.2

Data are means ± SD

SDS, standard deviation score

### Glucose control

**Fig. 2:**
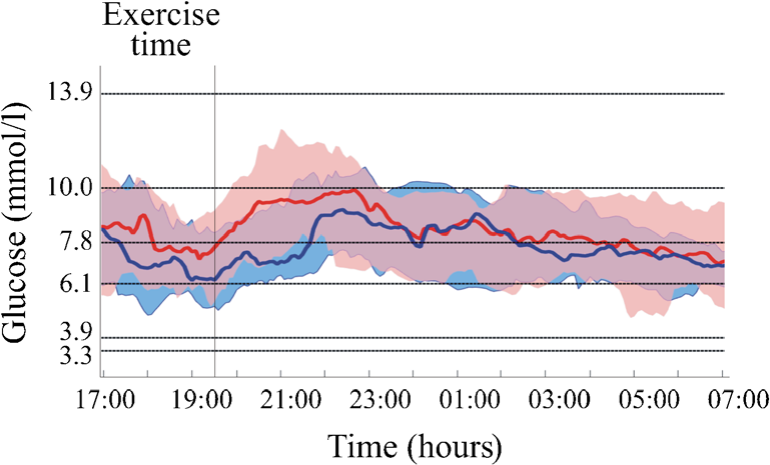
Median (IQR) sensor glucose during closed-loop (blue) and open-loop (red) insulin delivery, from exercise period (17:00 h) until morning (07:00 h), with standard glucose outcome measures [22] and common glucose target value of 6.1 mmol/l

Data representing glucose control are shown in Table 2, Fig. 2 and ESM Fig. 1. For the total duration of the study, we obtained 96% of sensor data during closed-loop delivery and 97% during open-loop (control) delivery. Data from all participants were included in the analysis. The median (interquartile range) proportion of time spent in hypoglycaemia below 3.3 mmol/l during the afternoon exercise and the night after, based on sensor glucose readings, was 0.00% for both groups (0.00–0.76% for closed-loop and 0.00–1.06% for open-loop, *p* = 0.7910). During the study, six hypoglycaemic events were recorded in the closed-loop group and 12 in the open-loop group (*p* = 0.5156); participants received rescue carbohydrates on seven occasions in the closed-loop group (total of 105 g) and on eight occasions in the open-loop group (total of 120 g) (Table 3).

**Table 2:** Comparison of glucose control during closed-loop and open-loop (control) insulin delivery

Variable	Closed-loop	Open-loop	*p* value
Time spent at low glucose			
Proportion of time spent with glucose below 3.3 mmol/l (%)			
All	0.0 (0.0–0.8)	0.0 (0.0–1.1)	0.7910
Night	0.0 (0.0–0.0)	0.0 (0.0–0.5)	0.9375
55%	0.0 (0.0–0.0)	0.0 (0.0–0.4)	0.6250
55/80%	0.0 (0.0–0.9)	0.0 (0.0–1.6)	0.5703
Proportion of time spent with glucose below 3.9 mmol/l (%)			
All	0.8 (0.0–3.2)	1.1 (0.0–4.1)	0.3811
Night	0.0 (0.0–3.5)	0.0 (0.0–2.8)	0.7910
55%	0.0 (0.0–0.0)	0.2 (0.0–4.5)	0.1909
55/80%	1.1 (0.0–3.4)	0.0 (0.0–3.3)	0.8394
Glucose AUC below 3.3 mmol/l (mmol/l × min)			
All	0.0 (0.0–7.0)	0.0 (0.0–5.7)	0.3394
Night	0.0 (0.0–0.0)	0.0 (0.0–1.4)	0.9375
55%	0.0 (0.0–0.0)	0.0 (0.0–0.3)	0.6953
55/80%	0.0 (0.0–1.3)	0.0 (0.0–5.7)	0.6523
Glucose AUC below 3.9 mmol/l (mmol/l × min)			
All	9.2 (0.0–80.2)	8.6 (0.0–33.2)	0.6874
Night	0.0 (0.0–8.3)	0.0 (0.0–12.0)	0.6221
55%	0.0 (0.0–0.0)	0.1 (0.0–12.2)	0.4548
55/80%	2.9 (0.0–17.4)	0.0 (0.0–24.9)	0.8394
LBGI			
All	0.4 (0.2–0.8)	0.4 (0.0–1.0)	0.6580
Night	0.3 (0.0–0.9)	0.0 (0.0–1.4)	0.8313
Time spent within target range			
Proportion of time spent with glucose within 3.9–10 mmol/l (%)			
All	84.1 (70.0–85.5)	68.7 (59.0–77.7)	0.0057
Night	92.8 (69.8–98.4)	73.3 (61.3–84.2)	0.0079
55%	80.9 (64.3–92.2)	68.1 (59.1–83.6)	0.0930
55/80%	75.3 (66.6–92.9)	68.4 (52.1–77.2)	0.0206
Proportion of time spent with glucose within 4.4–6.7 mmol/l (%)			
All	26.5 (16.5–35.2)	18.3 (8.2–28.5)	0.0111
Night	17.0 (9.7–38.5)	15.5 (0.0–32.9)	0.0479
55%	22.1 (7.0–51.6)	15.4 (3.9–30.7)	0.0793
55/80%	23.0 (11.4–35.8)	14.8 (8.3–29.3)	0.0674
Median glucose (mmol/l)			
All	7.5 (7.1–8.1)	8.6 (7.3–9.2)	0.0089
Night	7.8 (7.0–8.4)	8.1 (7.0–9.5)	0.1044
55%	7.8 (6.6–8.6)	8.7 (6.6–9.6)	0.0966
55/80%	7.7 (6.9–8.3)	8.5 (7.4–9.7)	0.0149
Mean glucose (mmol/l)^a^			
All	7.9 (7.4–8.6)	8.8 (7.5–9.5)	0.0228
Night	7.8 (6.8–8.4)	8.6 (7.5–9.8)	0.0152
55%	8.0 (6.8–8.8)	8.7 (6.6–9.6)	0.2790
55/80%	7.9 (7.2–8.8)	9.3 (7.5–9.9)	0.0057
Mean SD of glucose concentration^a^	41.1 (36.1–46.7)	44.6 (36.2–52.2)	0.1789
Time spent at high glucose			
Proportion of time spent with glucose above 13.9 mmol/l (%)			
All	1.3 (0.0–4.9)	2.2 (0.0–11.9)	0.0103
Night	0.0 (0.0–0.0)	0.0 (0.0–5.2)	0.1484
55%	0.0 (0.0–1.5)	0.0 (0.0–7.2)	0.0420
55/80%	2.3 (0.0–6.7)	1.1 (0.0–17.2)	0.0227
Proportion of time spent with glucose above 10 mmol/l (%)			
All	14.6 (12.1–29.5)	28.1 (16.5–39.2)	0.0228
Night	7.2 (0.2–23.9)	22.7 (9.1–38.7)	0.0156
55%	17.1 (7.2–33.0)	25.2 (6.0–39.3)	0.3144
55/80%	20.8 (5.0–28.9)	29.6 (17.6–45.4)	0.0228
Glucose AUC above 13.9 mmol/l (mmol/l × min)			
All	17.3 (0.0–225.2)	46.1 (0.0–564.7)	0.2293
Night	0.0 (0.0–0.0)	0.0 (0.0–124.4)	0.0547
55%	0.0 (0.0–15.4)	0.0 (0.0–103.4)	0.1475
55/80%	11.4 (0.0–107.0)	1.3 (0.0–293.8)	0.0681
AUC above 10 mmol/l (mmol/l × min)			
All	2011.6 (436.5–6006.4)	2222.0 (747.0–5575.6)	0.3905
Night	205.1 (0.0–3093.8)	11,226.9 (2653.4–20,212.0)	0.0003
55%	224.2 (99.8–660.9)	539.1 (74.4–1089.9)	0.3341
55/80%	606.9 (38.1–1029.8)	808.5 (359.8–1852.9)	0.0152
HBGI			
All	3.6 (2.9–5.6)	6.2 (3.4–7.9)	0.0080
Night	3.2 (1.0–4.4)	5.5 (2.8–7.3)	0.0051
Fasting glucose (mmol/l), all	6.8 (6.1–8.7)	7.3 (6.7–8.3)	0.2431

Data are shown as median (IQR)

^a^Nonparametric analyses for data on glucose control (paired nonparametric Wilcoxon signed rank test) and outcome data are presented as median (IQR) although variables for the analyses were presented as mean (glucose, glucose concentration SD)

All: whole study period from 15:00 h on day of exercise until 13:00 h on the following day; Night: period from 22:00 h on day of exercise until 07:00 h on the following day

55%, exercise protocol with moderate physical activity; 55/80%, exercise protocol with moderate physical activity and high-intensity sprints; HBGI, high blood glucose index; LBGI, low blood glucose index

**Table 3:** Hypoglycaemic events, rescue carbohydrate requirement and ketonuria events during closed-loop and open-loop (control) insulin delivery

Event	Closed-loop	Open-loop	*p* value
Hypoglycaemia			
All	6	12	0.5156
Night	3	4	1.0000
Rescue carbohydrates needed			
No. of occasions	7	8	
Total quantity given (g)	105	120	
Ketonuria, all	0	1	

Data are presented as number of events unless stated otherwise

All: whole study period from 15:00 h on day of exercise until 13:00 h on the following day; Night: period from 22:00 h on day of exercise until 07:00 h on the following day

Closed-loop delivery of insulin increased the proportion of time spent within the target glucose range of 3.9–10 mmol/l when compared with open-loop delivery: 84.1% (70.0–85.5) vs 68.7% (59.0–77.7), *p* = 0.0057 (Table 2). This was also true when calculated for the night alone: 92.8% (69.8–98.4) for closed-loop delivery and 73.3% (61.3–84.2) for open-loop delivery, *p* = 0.0079. The overall median and mean glucose levels were lower during closed-loop delivery of insulin than during open-loop delivery (*p* = 0.0089 and *p* = 0.0228, respectively). This was achieved with a significantly lower total amount of insulin delivered using the closed-loop device (112.6 U vs 203.7 U with open-loop, *p* = 0.0123), on account of less basal insulin (80.3 U vs 155.2 U with open-loop, *p* = 0.0074), with no difference in bolus insulin delivered (36.4 U via closed-loop and 40.3 U via open-loop, *p* = 0.8228) (Table 4). Similarly, closed-loop delivery of insulin increased the proportion of time spent within glucose range 4.4–6.7 mmol/l when compared with open-loop delivery (26.5% vs 18.3%, *p* = 0.0111) (Table 2).

**Table 4:** Comparison of insulin delivery via closed-loop and open-loop devices over whole period

Insulin delivered	Closed-loop	Open-loop	*p* value
Total daily insulin (U)	112.6 (73.1–200.3)	203.7 (91.6–277.1)	0.0123
Bolus insulin (U)	36.4 (27.6–55.6)	40.3 (31.2–48.3)	0.8228
Basal insulin (U)	80.3 (42.5–152.9)	155.2 (58.5–235.6)	0.0074

Data are shown as median (IQR)

Whole study period was from 15:00 h on day of exercise until 13:00 h on the following day

During closed-loop delivery of insulin, participants spent less time in the glucose range above 10.0 mmol/l (*p* = 0.0228 vs closed-loop) and above 13.9 mmol/l (*p* = 0.0103 vs closed-loop) (Table 2). High blood glucose index was lower when closed-loop delivery was used (3.6 vs 6.2, *p* = 0.0080) (Table 2).

There were no significant differences for other variables of hypoglycaemia (i.e. proportion of time spent with glucose below 3.9 mmol/l, AUC below 3.3 mmol/l and 3.9 mmol/l, and low blood glucose index). There was no difference in glucose variability between treatment modalities as measured by mean SD (Table 2).

SMBG data on glucose control during exercise and early recovery time are shown in Table 5 and data on sensor glucose analysis in Table 6. Active insulin amount at the beginning of the exercise and blood glucose values at the beginning and at the end of the exercise were similar for both treatment modalities. The difference between blood glucose levels at the start and end of exercise was − 2.6 (− 4.6 to − 1.6) mmol/l for closed-loop insulin delivery and − 2.2 (− 3.5 to − 0.9) mmol/l for open-loop delivery (*p* = 0.1000). During exercise, there was one hypoglycaemic event in the closed-loop group compared with four in the open-loop group.

**Table 5:** SMBG values during the exercise period and for 2 h after

Period	Blood glucose (mmol/l)		*p* value
	Closed-loop	Open-loop	
Physical activity start	8.3 (7.4–11.7)	9.2 (7.3–10.8)	0.9923
Start + 15 min	8.2 (6.5–10.9)	8.9 (7.1–10.4)	0.6967
Start + 30 min	7.0 (4.7–8.6)	7.5 (5.6–9.4)	0.5316
Physical activity end	5.8 (4.3–8.0)	7.2 (4.9–8.7)	0.2039
Δ Start to end	−2.6 (−4.6 to −1.6)	−2.2 (−3.5 to −0.9)	0.1000
End + 30 min	6.3 (5.2–7.6)	8.4 (5.7–10.4)	0.0093
End + 60 min	7.0 (5.7–8.4)	9.5 (6.7–11.6)	0.0006
End + 90 min	8.0 (6.6–9.5)	10.4 (9.2–13.7)	0.0001
End + 120 min	8.5 (7.3–10.5)	11.0 (8.0–14.6)	0.0003

Data are shown as median (IQR)

Δ Start to end: change in glucose levels at the end compared with starting level

**Table 6:** Sensor glucose analysis for the exercise period and for 4 h after

Variable	Closed-loop	Open-loop	*p* value
Proportion of time spent with glucose below 3.3 mmol/l (%)	0.0 (0.0–1.0)	0.0 (0.0–0.0)	0.5781
Proportion of time spent with glucose below 3.9 mmol/l (%)	0.0 (0.0–4.2)	0.0 (0.0–0.5)	0.9023
Proportion of time spent with glucose within 3.9–10 mmol/l	83.9 (68.2–93.2)	62.5 (39.1–79.7)	0.0012
Proportion of time spent with glucose above 10 mmol/l	10.4 (3.1–28.6)	33.2 (12.0–53.6)	0.0055
Proportion of time spent with glucose above 13.9 mmol/l	0.0 (0.0–0.0)	0.0 (0.0–23.4)	0.0078
Median glucose (mmol/l)	7.2 (6.5–8.6)	8.6 (7.5–10.3)	0.0251
Mean glucose (mmol/l)	7.4 (6.7–8.7)	8.8 (7.8–10.8)	0.0152
No. of hypoglycaemia events	1	4	0.5000
Active insulin at start (U)	6.2 ± 4.7	5.6 ± 3.0	0.3641

Data are shown as median (IQR) or means (± SD)

Between 7:00 h and 13:00 h on the day following physical activity (Table 7), the proportion of time spent in hypoglycaemia was low in both study arms (0.0% for both study groups, *p* = 0.1094). The difference between the study arms in the proportion of time spent with glucose within the range 3.9–10 mmol/l favouring closed-loop delivery of insulin did not reach statistical significance (72.8% in closed-loop vs 65.5% in open-loop, *p* = 0.0569).

**Table 7:** Glucose control on the day after physical activity (between 7:00 h and 13:00 h)

Variable	Closed-loop	Open-loop	*p* value
Proportion of time spent with glucose below 3.3 mmol/l	0.0 (0.0–0.0)	0.0 (0.0–0.3)	0.1094
Proportion of time spent with glucose below 3.9 mmol/l	0.0 (0.0–0.0)	0.0 (0.0–6.5)	0.0273
Proportion of time spent with glucose within 3.9–10 mmol/l	72.8 (57.8–83.7)	65.5 (54.3–80.5)	0.0569
Proportion of time spent with glucose above 10 mmol/l	26.5 (16.3–40.4)	28.9 (12.1–45.7)	0.3135
Proportion of time spent with glucose above 13.9 mmol/l	0.0 (0.0–7.7)	0.0 (0.0–10.6)	0.4316
Median glucose (mmol/l)	8.7 (7.8–9.5)	8.6 (7.6–10.2)	0.7652
Mean glucose (mmol/l)	8.5 (7.1–8.8)	8.5 (7.4–9.8)	0.2957

Data are shown as median (IQR)

**Fig. 3:**
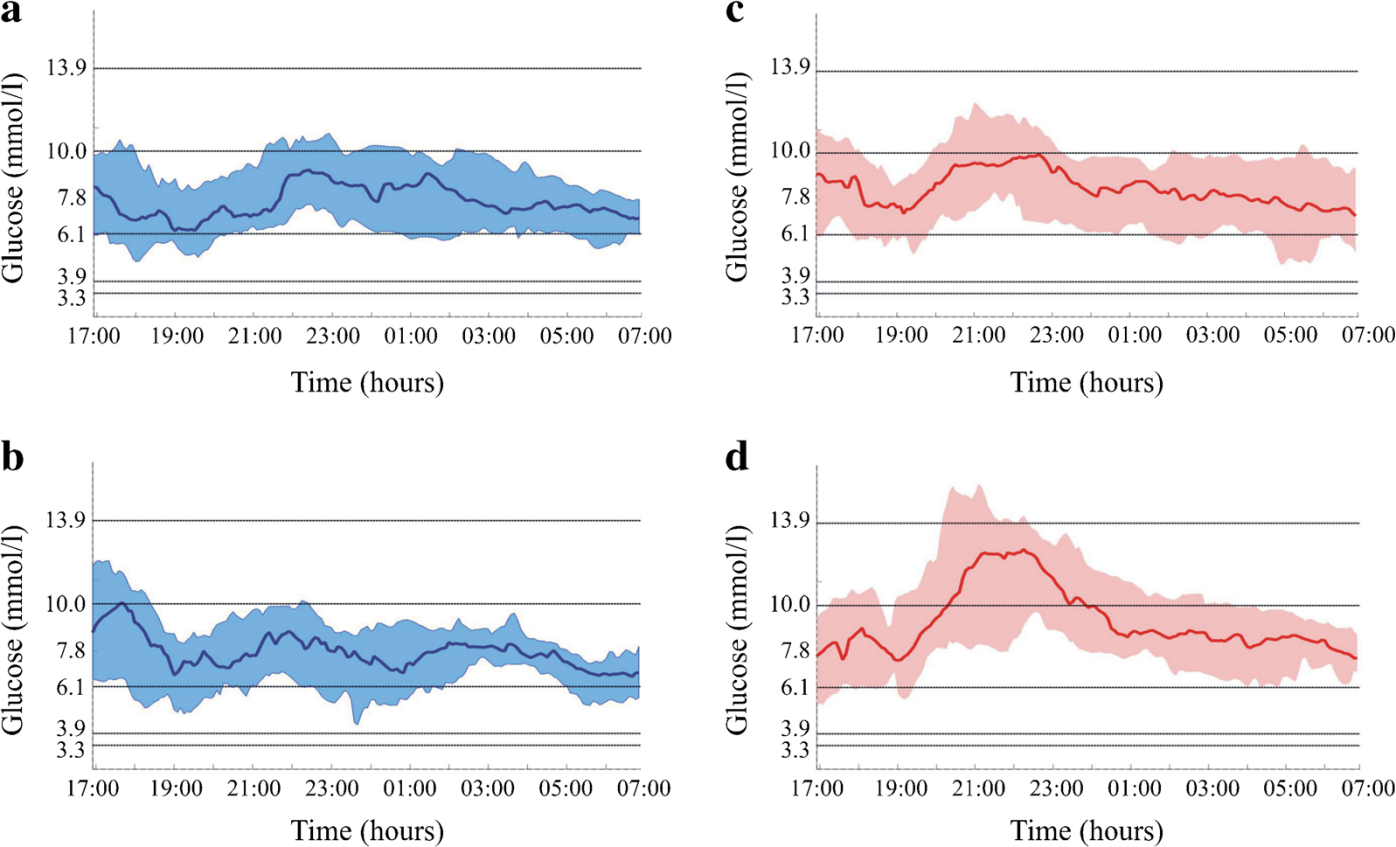
Median (IQR) sensor glucose during closed-loop (blue, **a**, **b**) and open-loop (red, **c**, **d**) insulin delivery, from exercise period (17:00 h) until morning (07:00 h), for 55% $\overset{\cdot }{V}{\mathrm{O}}_{2\max }$ moderate physical activity protocol (**a**, **c**) and 55/80% $\overset{\cdot }{V}{\mathrm{O}}_{2\max }$ moderate physical activity with high-intensity sprints (**b**, **d**) with consensus glucose outcome measures [22] and mean glucose target value 6.1 mmol/l

In the subanalysis comparing the effects of the different physical activity protocols on the time spent at various blood glucose concentrations, we observed significant improvement in the proportion of time spent within the range 3.9–10 mmol/l (*p* = 0.0206), time spent above 13.9 mmol/l (*p* = 0.0227) and median glucose value (*p* = 0.0149) when closed-loop delivery of insulin was used during the 55/80% $\overset{\cdot }{V}{\mathrm{O}}_{2\max }$ protocol (Table 2, Fig. 3) but not during the 55% $\overset{\cdot }{V}{\mathrm{O}}_{2\max }$ protocol.

### Adverse events

No serious adverse events or severe hypoglycaemia occurred during the study. One participant experienced ketonuria between 1 and 5 mmol/l (Keto-Diabur Test 5000, Roche, Switzerland) 1 h before exercise during the open-loop delivery of insulin on the 55% $\overset{\cdot }{V}{\mathrm{O}}_{2\max }$ visit, which was associated with antecedent set occlusion and hyperglycaemia. One participant had a local skin reaction to the subcutaneous glucose sensor adhesive. Another participant broke his wrist playing basketball on the day before his last study visit but was able to follow the study protocol.

## Discussion

The results of this study show that the use of closed-loop insulin delivery is safe during and after unannounced physical activity, either moderate or mixed moderate with periodic sprints. Closed-loop delivery substantially increased the time spent within normal blood glucose range, without an increased risk of hypoglycaemia, and reduced the time spent in hyperglycaemia.

This is, to our knowledge, the first randomised controlled study that investigates the efficacy of closed-loop insulin delivery with unannounced physical activity and without uncovered snacks in a juvenile population with type 1 diabetes. It is also the first study to incorporate a physical activity protocol with high-intensity sprints which resembles everyday activities of young people. The scenario of not announcing physical activity to the system and not providing uncovered snacks relates to real-life situations experienced by an adolescent population often unable to adhere to the recommended measures before physical activity [2, 8].

The primary endpoint of the study (difference in time spent in hypoglycaemia below 3.3 mmol/l) was not met. The reason for this was probably related to the protocol design. Due to meticulous adherence to the in-hospital standard operating procedure for preventing hypoglycaemia, implemented by trained nurse educators during in-hospital stay, there was consequently an equally short time spent in hypoglycaemia in both study groups.

Previous studies demonstrated that the use of SAPs with a predictive low-glucose insulin-suspend (PLGS) function could reduce the risk of hypoglycaemia after physical activity [26]. The use of these pumps in the paediatric population also reduces the risk of nocturnal hypoglycaemia [27]. The overnight hypoglycaemia event rate was comparably low with both closed-loop and open-loop insulin delivery devices in the present study, and the amount of time spent in hypoglycaemia below 3.3 mmol/l or below 3.9 mmol/l was very low.

We observed a significant increase in the proportion of time spent within glucose target range during closed-loop use; less insulin was delivered due to reduced basal insulin delivery. Regardless of physical activity protocol, the closed-loop device reduced blood excursions during the night, with 93% of the time spent within the glucose target range of 3.9–10 mmol/l. Contrary to this, the automated insulin-suspend function in commercial SAPs has a limited effect on the total time spent within the normal range [27], because this function is not designed to respond to higher insulin requirements during hyperglycaemia. Hyperglycaemia is often a consequence of higher-intensity exercise and reduced time spent in hyperglycaemia may be important for preventing damage to the developing brain [28].

The diversity of physical activity represents an important hindrance towards fully automated 24/7 closed-loop insulin delivery due to numerous factors influencing glucose control, such as duration of activity, intensity, time from previous and time to next meal, insulin on board and physical capacity [8]. We observed a consistent decline in blood glucose during physical activity, regardless of treatment modality, with a comparable amount of active insulin at the beginning of the activity. The glucose levels at the beginning of unannounced physical activity were postprandial (following a snack) and in most instances were within high normal range. This is in line with the recent recommendations for starting blood glucose level before physical activity [8].

Providing additional information to the system (e.g. heart rate), exercise announcement or adding additional simple carbohydrates can be of benefit in the intermediate stage of development of artificial pancreas [17, 29, 30]. In a recent randomised crossover trial with an afternoon mixed exercise protocol in one arm, the closed-loop device switched to exercise algorithm when triggered by a portable heart rate monitor (HRM) [17]. The number of hypoglycaemic events was low and comparable in both arms, but the HRM-informed algorithm improved the time spent with glucose levels below 3.9 mmol/l. The glucose variables with the HRM signal in use are comparable with those in our present data: the overall proportion of time spent below 3.9 mmol/l and within the range 3.9–10 mmol/l are reported as 3.2% and 77%, respectively [17]. In the present study, exercise was unannounced to the system in the closed-loop arm and no precautionary measures were made before exercise, as this resembles real-life situations. For the time during and after exercise in the open-loop arm, we incorporated adjustments in insulin therapy based on ISPAD guidelines. Without reduction in basal rates after exercise there would probably be significantly more episodes of hypoglycaemia in the open-loop control group. The present study tested whether the closed-loop algorithm can prevent hypoglycaemia during and after an unannounced physical activity. The data showed that there is no need to announce physical activity to the system. However, a more prolonged or vigorous physical activity might benefit from an announcement.

The addition of glucagon in the dual-hormone (insulin and glucagon) system can further reduce hypoglycaemia but limited data exist so far to quantify the improved safety compared with the single-hormone system with announced physical activity. A recent study in adults compared the two systems and showed improved time spent below a glucose level of 4 mmol/l when using the dual-hormone system, with a low hypoglycaemic event rate in both study arms [18]. The proportion of time spent within the range 4–10 mmol/l was moderately higher than our findings for 3.9–10 mmol/l, and the proportion of time spent with glucose levels below 4 mmol/l was similar to our findings for 3.9 mmol/l, but this comes at the expense of increased cost and device complexity in the dual-hormone system.

One limitation of the present study was that it was conducted in a well controlled in-hospital environment. Free-living studies with competition–motivation are needed, as are studies where blood glucose levels are lower at the beginning of the physical activity. The relatively small sample size increases the possibility of type 1 error and is another limitation of the study. Furthermore, most of the participants were regularly active in early afternoons, and high-risk individuals with hypoglycaemia unawareness or those who recently experienced an episode of more severe hypoglycaemia were excluded; therefore, a generalisation of these data to the general population of young people with type 1 diabetes may be difficult. Participants received instructions on meals for the day before the intervention but we did not check compliance.

In conclusion, the present study demonstrated that closed-loop insulin delivery was safe and efficiently increased the time spent within the desired glucose range, with less insulin delivered and without an increase in hypoglycaemia during and after unannounced physical activity in adolescents with type 1 diabetes. Larger studies using closed-loop insulin delivery during physical activity in free-living conditions and during competitive sports are warranted, as well as studies incorporating high-risk individuals who could especially benefit from the hypoglycaemia risk reduction.

## Electronic supplementary material

ESM Fig. 1 (PDF 11027 kb)

## Abbreviations

HRM: Heart rate monitor
IQR: Interquartile range
ISPAD: International Society for Paediatric and Adolescent Diabetes
SAP: Sensor-augmented insulin pump
SMBG: Self-monitoring of blood glucose
