# Supplementary material for: Closed-loop glucose control in young people with type 1 diabetes during and after unannounced physical activity: a randomised controlled crossover trial
Source: Diabetologia. 2017 Aug 24;60(11):2157–67. doi: 10.1007/s00125-017-4395-z (PMC6448906; doi:10.1007/s00125-017-4395-z)
Supplement: Supplementary file 1 — (PDF 11027 kb) [file 125_2017_4395_MOESM1_ESM.pdf]

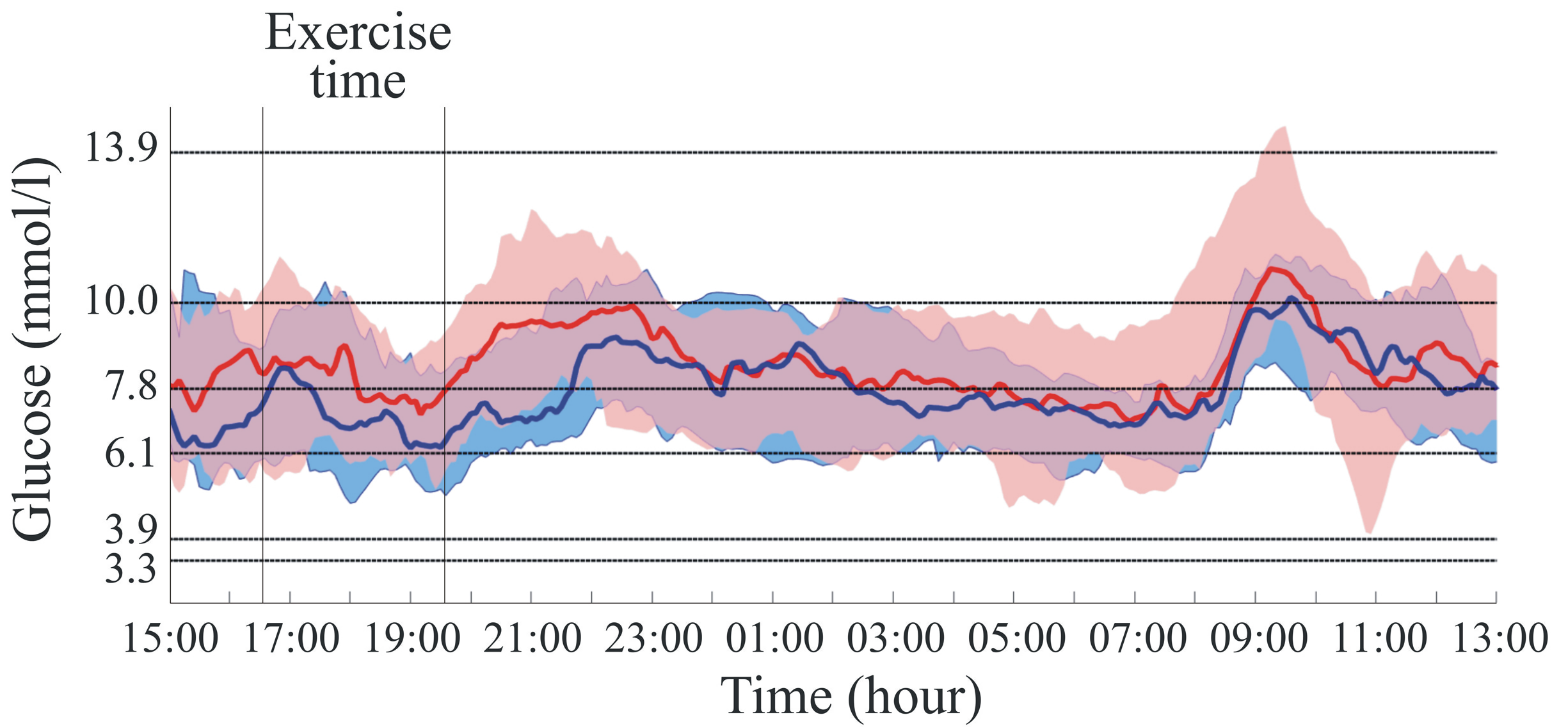

ESM Figure 1. Median sensor glucose for the whole observational period
Median (IQR) sensor glucose during closed-loop (blue) and open-loop (red) insulin delivery, from beginning (15:00) till the end (13:00) of observational period.
